# The Alzheimer’s disease-associated protective Plcγ2-P522R variant promotes immune functions

**DOI:** 10.1186/s13024-020-00402-7

**Published:** 2020-09-11

**Authors:** Mari Takalo, Rebekka Wittrahm, Benedikt Wefers, Samira Parhizkar, Kimmo Jokivarsi, Teemu Kuulasmaa, Petra Mäkinen, Henna Martiskainen, Wolfgang Wurst, Xianyuan Xiang, Mikael Marttinen, Pekka Poutiainen, Annakaisa Haapasalo, Mikko Hiltunen, Christian Haass

**Affiliations:** 1 Institute of Biomedicine, University of Eastern Finland, Kuopio, Finland; 2 Deutsches Zentrum für Neurodegenerative Erkrankungen (DZNE), München, Munich, Germany; 3 Institute of Developmental Genetics, Helmholtz Zentrum München, Munich, Germany; 4 Metabolic Biochemistry, Biomedical Center (BMC), Faculty of Medicine, Ludwig-Maximilians-Universität München, Munich, Germany; 5 A.I. Virtanen Institute for Molecular Sciences, University of Eastern Finland, Kuopio, Finland; 6 Munich Cluster for Systems Neurology (SyNergy), Munich, Germany; 7 Structural and Computational Biology Unit, European Molecular Biology Laboratory, Heidelberg, Germany; 8 Center of Diagnostic Imaging, Department of Cyclotron and Radiopharmacy, Kuopio University Hospital, Kuopio, Finland

**Keywords:** Alzheimer’s disease, Macrophage, Microglia, Knock-in mouse model, *PLCG2*

## Abstract

**Background:**

Microglia-specific genetic variants are enriched in several neurodegenerative diseases, including Alzheimer’s disease (AD), implicating a central role for alterations of the innate immune system in the disease etiology. A rare coding variant in the *PLCG2* gene (rs72824905, p.P522R) expressed in myeloid lineage cells was recently identified and shown to reduce the risk for AD.

**Methods:**

To assess the role of the protective variant in the context of immune cell functions, we generated a Plcγ2-P522R knock-in (KI) mouse model using CRISPR/Cas9 gene editing.

**Results:**

Functional analyses of macrophages derived from homozygous KI mice and wild type (WT) littermates revealed that the P522R variant potentiates the primary function of Plcγ2 as a Pip2-metabolizing enzyme. This was associated with improved survival and increased acute inflammatory response of the KI macrophages. Enhanced phagocytosis was observed in mouse BV2 microglia-like cells overexpressing human PLCγ2-P522R, but not in PLCγ2-WT expressing cells. Immunohistochemical analyses did not reveal changes in the number or morphology of microglia in the cortex of Plcγ2-P522R KI mice. However, the brain mRNA signature together with microglia-related PET imaging suggested enhanced microglial functions in Plcγ2-P522R KI mice.

**Conclusion:**

The AD-associated protective Plcγ2-P522R variant promotes protective functions associated with TREM2 signaling. Our findings provide further support for the idea that pharmacological modulation of microglia via TREM2-PLCγ2 pathway-dependent stimulation may be a novel therapeutic option for the treatment of AD.

## Findings

**Fig. 1:**
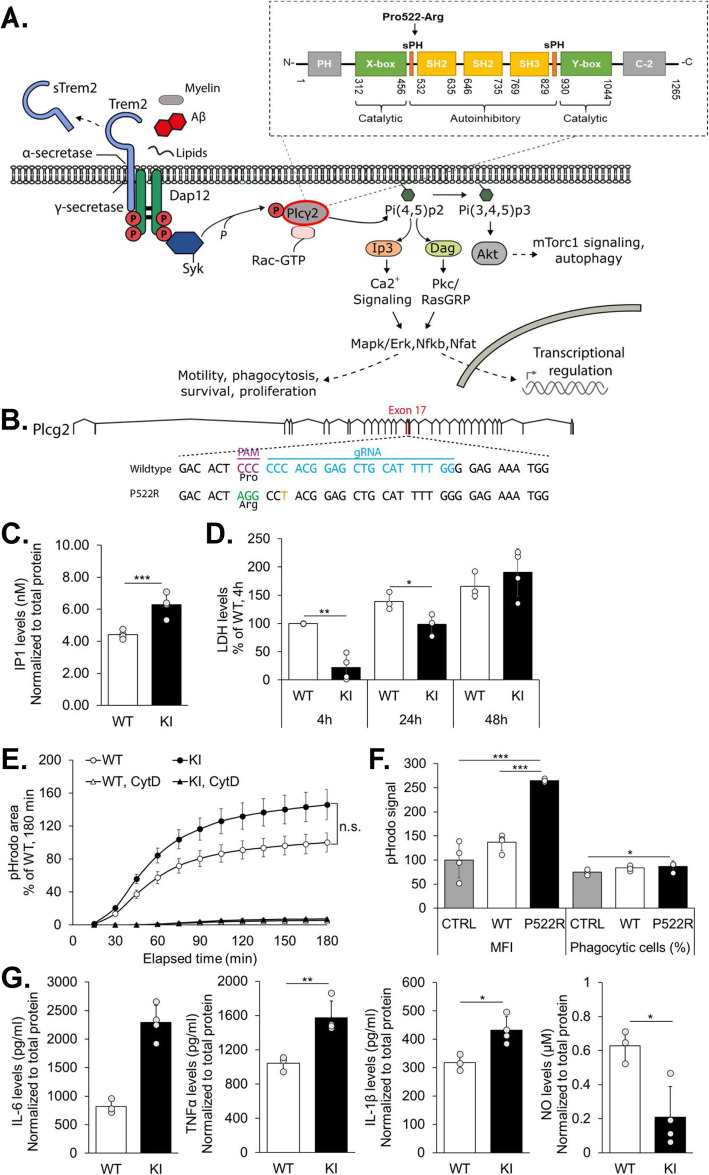
The Plcγ2*-*P522R variant increases Plcγ2 enzymatic activity, survival, phagocytic activity, and inflammatory response in macrophages. **a** A schematic view of Plcγ2 signaling. **b** Strategy to target murine *Plcγ2* locus, indicating protospacer region (blue), protospacer adjacent region (PAM, purple), and introduced nucleotide changes (missense: green; silent: orange). **c** Inositol monophosphate (Ip1) formation in Plcγ2-P522R knock-in (KI) and wild type (WT) bone marrow-derived macrophages (BMDMs). Values are normalized to the total protein concentration. Mean ± SD, *n* = 4 per genotype, 2 technical replicates. Two-way ANOVA, LSD post hoc test, ***p < 0.001. D) Lactate dehydrogenase (LDH) levels in KI and WT BMDMs after 4 h, 24 h and 48 h macrophage colony stimulation factor 1 (mCSF) withdrawal. Values are normalized to the maximum LDH release within each well. Mean ± SD, % of WT (4 h), *n* = 3–4 per genotype, 2 technical replicates. Two-way ANOVA, LSD post hoc test, *p < 0.05, **p < 0.01. E) Phagocytic activity in KI and WT BMDMs. Cytochalasin D (CytD, 5 μM) was used as a control. pHrodo signal is normalized to the cell count in each well. Mean ± SEM, % of WT (180 min), *n* = 6–7 per genotype, 3 technical replicates. Independent samples *t*-test, n.s. non-significant. F) Phagocytic activity in BV2 microglial cells overexpressing Myc-tagged human PLCγ2-P522R, PLCγ2-WT, or control (CTRL) vector shown as median fluorescence intensity (MFI, left panel) and percentage of phagocytic cells (right panel). Mean ± SD, % of CTRL, *n* = 4. Mann-Whitney U test, *p < 0.05, ***p < 0.001. G) Interleukin-6 (IL-6), tumor necrosis factor α (TNFα), IL-1β, and nitric oxide (NO) levels in conditioned media of KI and WT BMDMs upon 3 h lipopolysaccharide (LPS) and interferon gamma (IFNγ)-treatment. Values are normalized to the total protein concentration in the corresponding wells. Mean ± SD, *n* = 3–4 per genotype. Independent samples *t*-test, *p < 0.05, **p < 0.01

Recent genome-wide association studies have identified several Alzheimer’s disease (AD)-associated risk loci in genes selectively or preferentially expressed in microglia (e.g. *TREM2, ABI3, PLCG2*) [1]. A rare coding variant in the microglia−/macrophage-specific *PLCG2* gene (rs72824905, p.P522R, OR < 0.6) encoding phospholipase C gamma 2 (PLCγ2) enzyme was recently identified and shown to confer a reduced risk of AD [1]. Interestingly, the same *PLCG2* variant associated with a lower risk for other neurodegenerative diseases and increased the likelihood for longevity [2]. PLCγ2 catalyzes the conversion of phosphatidylinositol 4,5-bisphosphate (Pip2) to inositol 1,4,5-trisphosphate (Ip3) and diacylglycerol (Dag) upon activation of various transmembrane immune receptors. Ip3 and Dag regulate pathways related to e.g. survival, phagocytosis, and cytokine production via controlling intracellular calcium mobilization as well as protein kinase C, nuclear factor kappa-light-chain-enhancer of activated B cells (Nfκb), mitogen-activated protein kinase (Mapk/Erk), and protein kinase B (Akt) signaling [3–5] (Fig. 1a). The P522R variation locates in the regulatory domain of Plcγ2, but whether this variant affects the above-mentioned functions of microglia and other myeloid lineage cells, is not known. Based on recent studies in human induced pluripotent stem cell (iPSC)-derived PLCγ2 knockout microglia (iMG), PLCγ2 is required for the signaling pathway initiated by the Triggering receptor expressed on myeloid cells 2 (Trem2) (Fig. 1a) [6]. Loss of PLCγ2 function consequently reduces TREM2-dependent support of cell survival, phagocytosis and lipid metabolism. Since the protective PLCγ2-P522R variant reduces the risk for AD, we speculated that it may influence TREM2/PLCγ2-dependent signaling pathways in the opposite direction.

To address this question, we generated a Plcγ2-P522R knock-in (KI) mouse model using the CRISPR/Cas9 gene editing technology (Fig. 1b) [7, 8] to investigate the effects of the protective variant in isolated macrophages and in vivo in the mouse brain. Founder mice with successful incorporation of the variant and no off-target effects (Fig. S1) were used for generation of a homozygous Plcγ2-P522R line. Homozygous mice were viable, fertile, and did not show obvious detrimental phenotypes.

### The P522R variant increases Plcγ2 enzyme activity and enhances survival, phagocytosis, and inflammatory response

Bone marrow-derived macrophage (BMDM) cultures were established from *femur* and *tibia* bones of six-month-old Plcγ2-P522R KI mice and their wild type (WT) littermates [9–11]. The P522R variant has previously been shown to increase the production of inositol phosphate (Ip1, a surrogate of Ip3) in transiently transfected HEK293 and COS cells [12], while the loss of PLCγ2 or TREM2 in iMGs reduces Ip1 production [6]. Accordingly, significantly higher basal levels of Ip1 were detected in cultured KI BMDM cells as compared to WT cells (Fig. 1c), suggesting that the P522R variant exerts a mild hypermorphic effect on the basal enzyme activity of Plcγ2 in the cultured mouse BMDMs. The relatively mild increase of the catalytic activity may explain, why humans carrying the P522R variant apparently do not develop similar immune deficits that are associated with other hypermorphic *PLCG2* variants [3–5, 13]. The enhanced catalytic activity of Plcγ2 did not associate with the increased protein levels of Plcγ2 (Fig. S2A), suggesting that P522R variant directly affects catalysis probably via structural changes.

Next, we examined the functional outcomes of increased Plcγ2 activity in cultured BMDMs. In a recent study, compromised Ip3/Ip1 signaling due to PLCγ2 depletion was shown to interfere with cell survival, phagocytic activity, and inflammatory response in iMGs [6]. In contrast to the loss-of-function, the survival of the KI BMDMs was enhanced as compared to WT BDMDs after withdrawal of macrophage colony stimulation factor (m-CSF) (Fig. 1d). Similarly, a significant reduction in the activation of Caspase-3/7 was detected in KI BMDMs (Fig. S2B), suggesting that the P522R variant protects BMDMs from apoptosis. In line with these findings, Trem2 deficiency has been shown to compromise the survival of microglia and peripheral macrophages [11, 14, 15], whereas antibody-mediated stabilization of mature Trem2 strongly enhances the survival and proliferation of BMDMs [16, 17]. This further supports the notion that Trem2 and Plcγ2 act on the same pathway (Fig. 1a) [6], and that the Trem2 risk and Plcγ2 protective variants have opposite effects.

To further substantiate the opposite effects of the Plcγ2 protective variant and loss-of-function, we investigated if phagocytosis was enhanced by the protective variant. Although ~ 45% higher phagocytic activity was observed in the KI BMDMs as compared to the WT cells (Fig. 1e and Fig. S2C), this finding was not statistically significant. To address whether P522R variant enhances phagocytosis in microglia-like cells, cDNAs encoding human PLCγ2-WT and PLCγ2-P522R were expressed in BV2 cells (Fig. S2D). More than two-fold higher phagocytic activity was detected in BV2 cells overexpressing the PLCγ2-P522R variant as compared to those expressing PLCγ2-WT or a control plasmid (Fig. 1f). However, as compared to the control cells, the overall number of phagocytic cells was only 11 and 15% higher upon PLCγ2-WT and PLCγ2-P522R overexpression, respectively, suggesting an increased phagocytic capacity per cell. Although further functional studies focused on e.g. endosomal/lysosomal compartments are warranted in KI microglia, our results suggest that the P522R variant promotes the phagocytic capacity, which is consistent with reduced phagocytosis observed upon deletion of PLCγ2 [6].

Apart from Trem2-dependent signaling, Plcγ2 mediates inflammatory responses and cytokine production through Toll-like receptors [6, 18]. To assess an acute response of the BMDMs to an inflammatory stimulus, the cells were treated with lipopolysaccharide (LPS) and interferon-gamma (IFNγ). BMDMs from KI mice released significantly higher levels of tumor necrosis factor-α, (TNFα), interleukin-6 (IL-6), and -1β (IL-1β) into the culture medium (Fig. 1g), suggesting that macrophages expressing the P522R variant display a stronger and/or faster response to the stimulus. Despite higher cytokine secretion, significantly lower levels of nitric oxide (NO) were detected in the culture medium of KI BMDMs. The IFNγ-induced production of NO has been shown to exacerbate apoptosis and compromise cell viability in a variety of cell types, and the neurotoxic effect of activated microglia is largely mediated by NO [19–21]. Thus, our observation supports the idea that the P522R variant protects cells under different stress conditions.

### The mRNA profile associated with Plcγ2-P522R indicates microglia activation and the modulation of Plcγ2 signaling

**Fig. 2:**
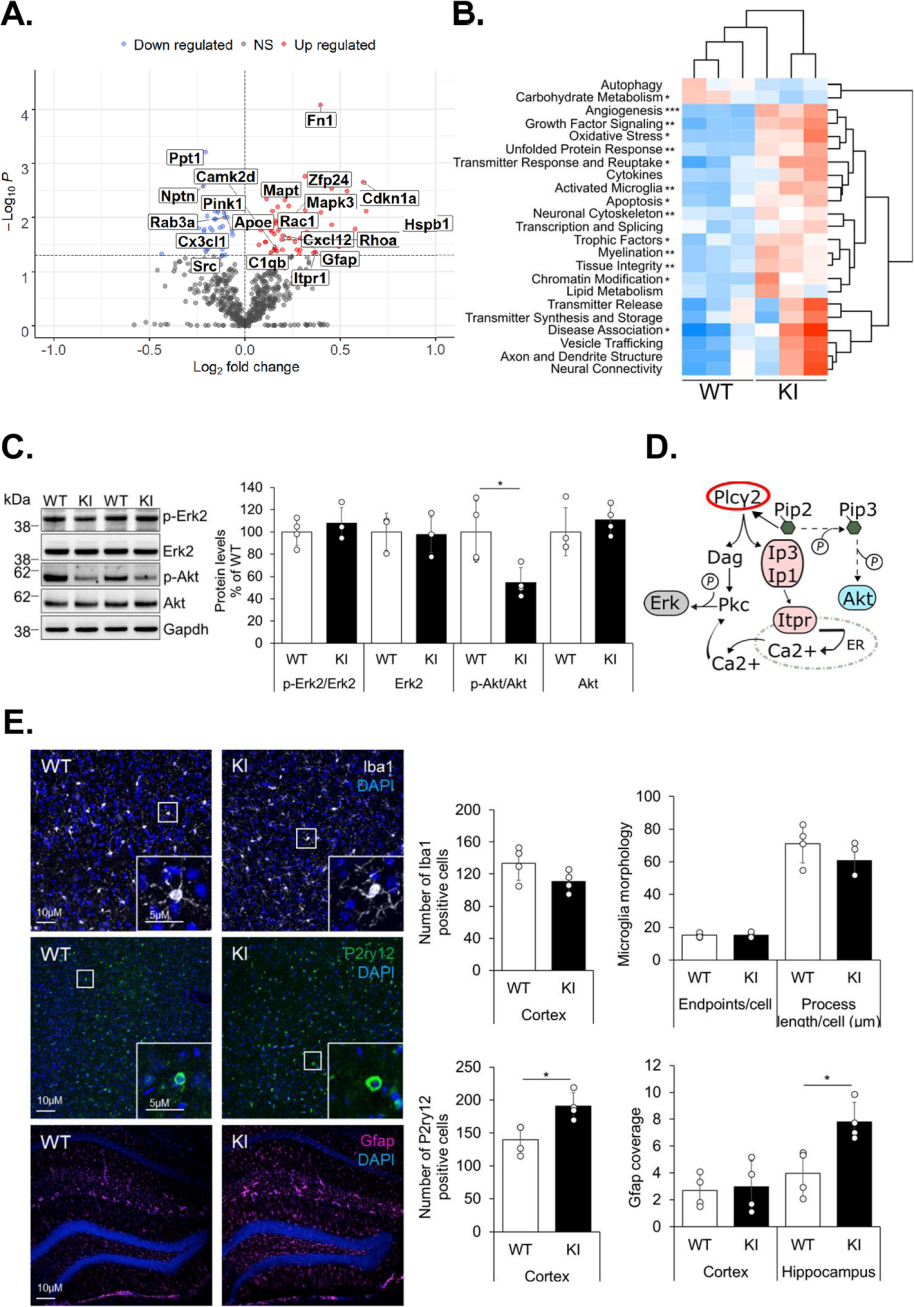
Activated microglia and astroglia in the brain of mice expressing the *Plcγ2-P522R* protective mutation. **a** Volcano blot showing significantly (*p* < 0.05 y-axis, dotted line) down- and upregulated genes and their log2-transformed fold-changes (x-axis) in the brain of Plcγ2-P522R homozygous knock-in (KI) mice as compared to the wild type (WT) littermates, *n* = 3 per genotype. **b** Heatmap showing pathway scores for neuropathology panel-derived pathway annotations. Significantly changed pathways are indicated with asterisk, *n* = 3 per genotype, Independent samples *t*-test, *p < 0.05, **p < 0.01, ***p < 0.001. **c** Representative images (left) of Iba1, P2ry12, and Gfap-stained cortex or hippocampus from WT and KI mice. Quantitation (right) of Iba1-positive microglia, microglia morphology, P2ry12, and Gfap levels. Scale bars represent 10 μm and 5 μm (box in the right corner). Mean ± SD, *n* = 4 per genotype, two-tailed unpaired *t*-test, *p < 0.05. D) Immunoblot showing phosphorylation status and total levels of Mapk/Erk and Akt in the total brain lysates of the WT and KI mice. Quantitation showing activity-related phosphorylation of Y204 and S473/474 epitopes in Mapk1/Erk2 and Akt (pan-antibody for Akt1 and Akt2), respectively, normalized to the respective total protein levels. Total protein levels are normalized to that of Gapdh within the same sample. Mean ± SD, % of WT, *n* = 3–4 per genotype. Independent samples t-test, *p < 0.05

Next, we searched for potential molecular changes in the brain of six-month old KI and WT male mice. Changes in total brain mRNA expression were determined utilizing a Nanostring neuropathology gene expression panel. Out of 770 analyzed genes, 57 (7%) were significantly up- and 32 (4%) significantly downregulated in the brain of KI as compared to WT mice (Fig. 2a, b**,** Fig. S3A). Notably, several genes directly linked to Plcγ2 signaling (e.g. *Itpr1, Camk2d, Mapk3/Erk1, Rac1*, and *Rhoa*) showed a significantly elevated expression in the KI mouse brain (Fig. S3B). *Itpr1* encodes an Ip3 receptor and acts directly downstream of Plcγ2. Similarly, Mapk/Erk-signaling is induced by Plcγ2 activation [4] and regulates pathways related to survival, proliferation, differentiation, and inflammatory responses in brain immune cells and other cell types [22]. Rac1 and RhoA can be activated via Trem2/Plcγ2-signaling [23–25] and play pivotal roles in microglia activation, migration, phagocytosis, and neuronal protection [25–29]. Thus, the higher expression of such targets could be directly associated with Plcγ2-dependent Ip3 signaling and is in line with improved survival, inflammatory response, and phagocytosis observed in KI macrophages.

To follow up the Nanostring-based findings in the brain tissue, including the potential Plcγ2-dependent signaling changes, we assessed the Mapk/Erk and Akt signaling pathways in the total brain lysates of six-month old mice. Activity-related phosphorylation of Mapk1/Erk2 (Y204) remained unaltered in the brain of KI mice, whereas the Mapk3/Erk1 phospho-protein was undetectable (Fig. 2c). However, activity-related phosphorylation of Akt1/2 (S473/474) was significantly reduced in the brain of KI mice (Fig. 2c). Activation of Akt is dependent on Pip3 (Fig. 1a), which is synthesized from Pip2, a substrate for Plcγ2. Therefore, this finding supports the notion that KI mice use more Pip2 as a substrate to produce Dag and Ip3 and consequently, less Pip3 exists for the activation of Akt1/2 (Fig. 2d). Akt modulates several pathways related to autophagy, survival, proliferation, and growth through Foxo, mTorC, and Gsk3β [30]. Accordingly to decreased Akt S473/474 phosphorylation, a trend towards a decrease in the inhibitory phosphorylation of Gsk3β (S9) was detected in KI mouse brain, while the ratio of LC3BII/I, a well-known index of autophagosomal activity, remained unaltered (Fig. S4A).

Based on a recent study, Plcγ2 is primarily expressed in microglia and at very low levels in other brain cell types [12]. Pathway analysis of the RNA data suggested higher microglia activation in the brain of KI as compared to the WT mice (Fig. 2b). Immunohistochemical analysis of the microglia marker Iba1 did not show significant alterations in microglia number or morphology in the brain of KI mice as compared to WT mice (Fig. 2e). The levels of a microglia-specific purinergic receptor P2ry12 were significantly higher in the cortex of KI mice as compared to WT mice (Fig. 2e), which is again in line with the finding that upon PLCγ2 KO in iMGs, the expression of P2RY12 is strongly diminished [6]. Although P2ry12 is a well-established homeostatic microglia marker [31], it has been considered also as a specific marker for healthy rodent CNS microglial cells [32] and is known to mediate microglia motility, phagocytosis of apoptotic cells, and protection of the blood-brain-barrier [33–35]. Also, the expression pattern analysis of P2RY12 in response to β-amyloid in aging human brain has suggested that P2RY12 expression by microglia should not be considered solely as a marker of resting microglia [36]. Collectively, our RNA and immunohistochemistry data suggest that the P522R variant promotes expressional changes linked to the altered activation status of microglia, which are not explained by the increased number of microglia. Recent transcriptomic studies in CNS immune cells have identified a unique subtype of disease-associated microglia (DAM), which is characterized by high expression of pro-survival, lipid metabolism, and phagocytosis-associated genes [31, 37]. Activation of DAM is driven in a Trem2/ApoE-dependent manner [31, 37] and it is essential for impeding amyloid seeding and formation of neuritic pathology [38]. To assess whether the protective variant influences the microglial activity status, expression of DAM signature genes was examined. Although the origin of cell type-specific expression cannot be confirmed, a significant upregulation of *Apoe,* one of the key upregulated DAM genes [31, 37, 39], was detected in the brain of KI mice (Fig. 2a). On the contrary, other DAM targets, such as *Trem2, Itgax,* and *Cd68,* remained unchanged. Additional qPCR-based analyses of microglia-specific DAM genes showed a trend towards increased expression of *Cst7, Tyrobp, Clec7a,* and *Ccl3* in the brain of KI mice (Fig. S3C). Taken together, the Plcγ2-P522R variant moderately increases at least a subset of DAM-associated signature genes. However, further studies focusing on isolated microglia from aged KI mice together with AD-associated pathology are needed to make firm conclusions related to DAM phenotype.

Apart from microglia, a significant upregulation of individual genes and pathways related to e.g. neuronal cytoskeleton and myelination was observed in the brain of the KI mice, suggesting that the protective variant exerts both cell autonomous and non-autonomous effects. Although no significant changes were detected in the levels of the pre- and post-synaptic markers Synaptophysin and Psd-95, comprehensive investigations related to microglia-mediated synaptic engulfment and the integrity of neural networks are needed in the future studies. Additionally, expression of the astrocytic marker, *Gfap*, was increased in the KI mouse brain (Fig. 2a). Accordingly, immunohistochemical analysis revealed a significantly stronger staining of Gfap and a hypertrophic morphology of astrocytes in the hippocampus of KI as compared to WT mice (Fig. 2c). These results suggest that the presence of the protective Plcγ2-P522R variant promotes a subset of reactive astrocytes in the KI mouse hippocampus.

### The Plcγ2-P522R protective variant increases 18F-FEPPA uptake in the brain of KI mice

**Fig. 3:**
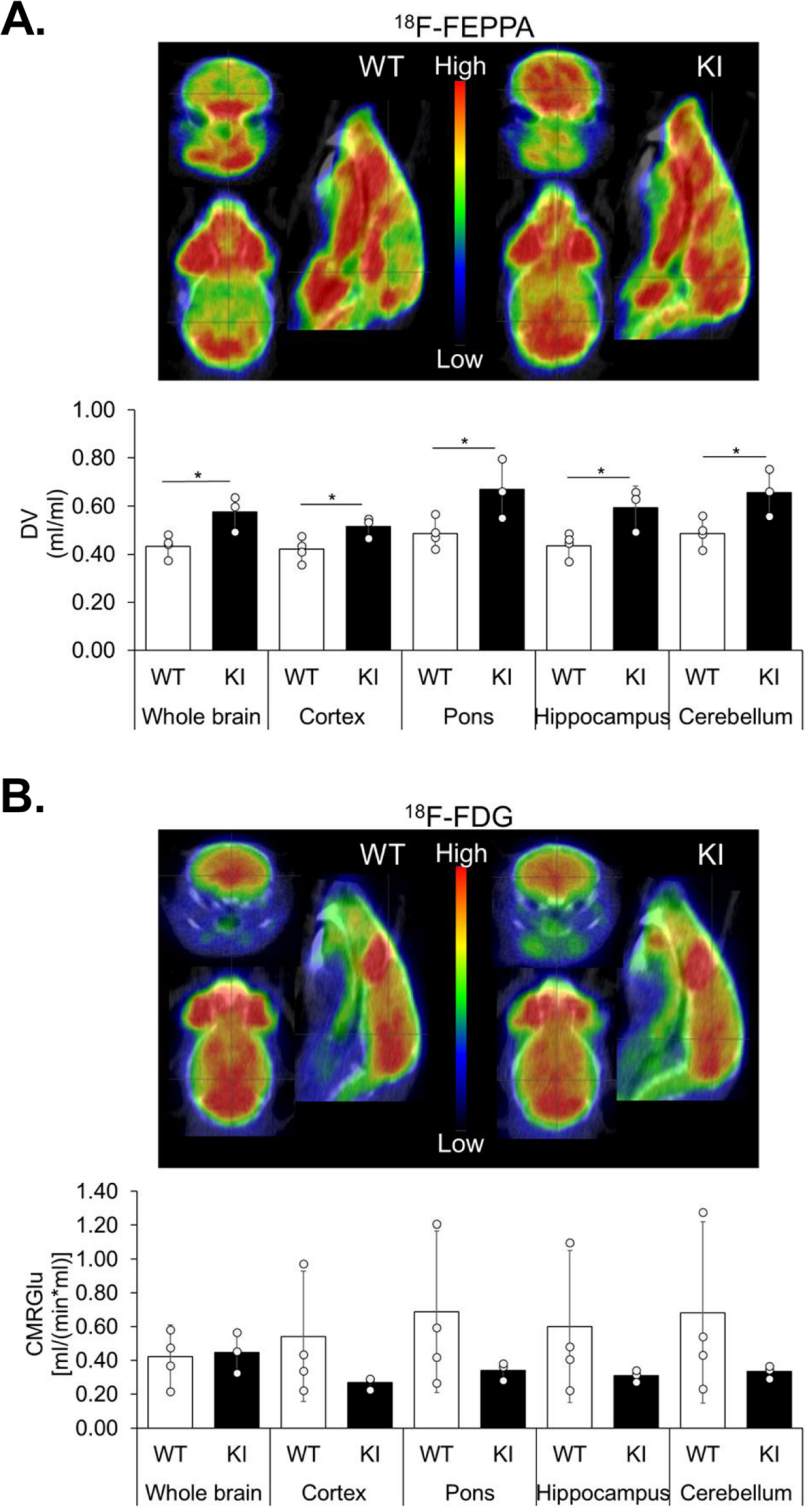
Plcγ2-P522R expression increases microglia activation in the brain of one-year-old mice. **a** Representative PET-images of the uptake of 18F-FEPPA (time window 15-70 min) (upper panel) in the brains of Plcγ2-P522R homozygous knock-in (KI) and wild type (WT) female mice at 1 year of age. Estimated total distribution volume (DV, ml/ml) in total brain, frontal cortex, pons, hippocampus, and cerebellum calculated with the Logan model using image derived input function (IDIF, lower panel), *n* = 3–4 per genotype. Independent samples *t*-test, *p < 0.05. **b** Representative PET images of the uptake of 18F-FDG (time window 20-80 min) in WT and KI mouse (upper panel). Cerebral metabolic rate of glucose [MRGlu, (ml/min*ml)] was estimated using the Patlak model (with lumped constant of 0.71, lower panel), *n* = 3–4 per genotype. Independent samples *t*-test

Finally, we elucidated the activation state of microglia in the Plcγ2-P522R KI mice in vivo by conducting PET imaging using the 18F-FEPPA radioligand specific for the translocator protein of 18 kDa (TSPO) [40]. According to the recent findings in *Trem2* KO mice, the TSPO signal is Trem2-dependent and therefore likely reflects changes in the activation state of microglia [11, 38, 40]. A significant increase in 18F-FEPPA signal was detected in one-year-old KI mice compared to WT female mice in all analyzed brain areas (Fig. 3a), which is in line with the opposite result in TREM2 loss-of-function mice [11, 38, 40]. Furthermore, impaired microglia activation has been shown to correlate with reduced cerebral glucose metabolism [11, 41]. On the other hand, microglial hyperactivation in *Grn* knockout mice also leads to reduced cerebral glucose metabolism [11, 41], demonstrating that both extremes of microglial activation states are deleterious. The cerebral uptake of glucose as detected by 18F-FDG-PET [42] did not show significant differences in glucose uptake in any of the brain regions studied nor any correlation between 18F-FEPPA and 18F-FDG signals in KI mouse brain, suggesting that the relatively mild microglia activation in the presence of the Plcγ2*-*P522R variant is not detrimental.

In conclusion, our results suggest that the protective Plcγ2-P522R variant potentiates the primary function of Plcγ2 and thus, promotes important immune cell functions in vitro*.* Based on the RNA, immunohistochemistry, and PET imaging data, Plcγ2-P522R also promotes microglia activation in vivo, without affecting the total number or morphology of microglia*.* Therefore, our findings suggest that the protective P522R variant sensitizes myeloid cells and potentially allows a more effective response upon neurodegeneration. The opposite phenotypes observed in *PLCG2* KO iMGs and Plcγ2-P522R KI mice also support the notion to therapeutically stimulate TREM2 functions [6, 16, 17, 43]. Moreover, the mild increase in the microglial functions due to the Plcγ2-P522R variant may instruct us to what extent we may activate microglia within a therapeutically safe window [16, 17, 41].

## Methods

### Animals

Plcγ2-P522R KI mice were generated by CRISPR/Cas9-assisted gene editing in mouse zygotes as described previously [7, 8, 11]. Briefly, pronuclear stage zygotes were obtained by mating C57BL/6 J males with superovulated C57BL/6 J females (Charles River). Embryos were then microinjected into the male pronucleus with an injection mix containing *Plcγ2*-specific CRISPR/Cas9 ribonucleoprotein (RNP) complexes. RNPs consisted of 50 ng/μl S.p. Cas9 HiFi protein (IDT), 0.6 μM crRNA (protospacer CCAAAATGCAGCTCCGTGGG; IDT), 0.6 μM tracrRNA (IDT), and 25 ng/μl mutagenic single-stranded oligodeoxynucleotide (ssODN) (5′ -CGCACTGGTCCTACTCTCCACCTTCTTGTGGAACCATTTCTCCCCAAAATGCAGCTCCGTAGGCCTAGTGTCCTGAGCCACAAGCATCCGAAAGGGCTTATTACAGCTCGCTCTGCCCTCTCCTGA-3′), comprising the P522R substitution and an additional silent mutation for genotyping purposes (IDT; see also Fig. 1b). After microinjection, zygotes were cultured in KSOM medium until they were transferred into pseudopregnant CD-1 foster animals. To identify putative off-target sites of the *Plcγ2*-specific guideRNA, the online tool CRISPOR (http://crispor.tefor.net/) was used. For analysis, genomic DNA of WT and heterozygous Plcγ2-P522R mice was isolated and predicted loci with a CFD score > 0.4 and an MIT score > 0.5 were PCR-amplified and Sanger sequenced. Animals without off-target mutations were used for generation of homozygous Plcγ2-P522R KI strain. Animals were raised and handled at the DZNE, Munich, and at the Laboratory Animal Center of the University of Eastern Finland, Kuopio. All animal experiments were carried out in accordance with the guidelines of the European Community Council Directives 86/609/EEC and approved by the Bavarian government and the Animal Experiment Board of Finland (ESAVI 21203–2019, EKS-004-2019).

### Mouse genotyping

Genomic DNA was purified from ear biopsies by isopropanol precipitation. The *Plcγ2* locus harboring the KI mutation was amplified by PCR using forward primer 5′-GCTGTCCTTCGGTGATGACA − 3′ and reverse primer 5′- CAGACCGCCTGTTGGGAATA − 3′. Resulting PCR products of 825 base pairs (bp) were further processed using the restriction enzyme StuI digesting the mutant KI allele into 412, 275, and 138 bp fragments and WT to 412 and 413 bp fragments. Fragments were subsequently detected from 1,5% agarose gel electrophoresis. The genotype of each mouse was verified by Sanger sequencing from the purified PCR product.

### Preparation of BMDM cultures

At the age of 6 months, KI male mice and their age- and gender-matched WT littermates were anesthetized with Ketamine-Xylazine mixture prior to trans-cardiac perfusion with ice cold saline (PBS). *Femur* and *tibia* bones were collected and processed for generation of BMDM cultures as described previously [9–11]. Briefly, bone marrow cells were flushed out using advanced RPMI 1640 medium (Life Technologies). Cells were differentiated for 7 days in advanced RPMI 1640 supplemented with 2 mM L-Glutamine, 10% (v/v)) heat-inactivated fetal calf serum (FCS), 100 U/ml penicillin, 100 μg/ml streptomycin and 50 ng/ml macrophage colony stimulation factor 1 (m-CSF) (R&D System) in non-cell culture treated dishes. After 7 days in culture, macrophages were carefully scraped, counted, and plated in optimal densities for subsequent analyses.

### Ip1 assay

Ip1 has been used as a standard readout when analyzing earlier identified *PLCγ2* variants in the previous studies [4, 6, 12]. To measure Ip1 formation, 50,000 KI (*n* = 4) and WT (*n* = 4) BMDMs per well were plated on a 96-well plate and allowed to attach overnight. IP-1 ELISA (Cisbio) assay was then used according to the manufacturer’s instructions. Ip1 concentration in each sample was calculated from the net optical density values using a standard curve. Values were normalized to the total protein concentration within each sample measured from the replicate wells. Data are normalized to the WT group and presented as mean ± SD.

### LDH cytotoxicity assay

For cytotoxicity assays, 10,000 KI (*n* = 4) and WT (*n* = 3) BMDMs per 96-well plate well were seeded in RPMI 1640 (without phenol red) supplemented with 2 mM L-Glutamine, 1% (v/v) heat-inactivated FCS, 100 U/ml penicillin, and 100 μg/ml streptomycin, and 50 ng/ml m-CSF. After the cells were attached, the medium was replaced with m-CSF-depleted medium. Medium samples from triplicate wells per sample were collected 4 h, 24 h and 48 h after m-CSF withdrawal. To determine maximum LDH release in each sample, replicate wells were treated with 2% Triton X-100 solution before medium collection. LDH cytotoxicity was measured according to the Cytotoxicity Detection Kit (LDH, Roche) protocol. LDH levels in each sample were normalized to the maximum LDH release in the corresponding sample. Data are normalized to the WT BMDMs and presented as mean ± SD.

### Caspase-3/7 activation assay (additional data)

For Caspase 3/7 activation assay, 10,000 KI (*n* = 3) and WT (*n* = 3) BMDMs per 96-well plate well were seeded in RPMI 1640 (without phenol red) supplemented with 2 mM L-Glutamine, 1% (v/v) heat-inactivated FCS, 100 U/ml penicillin, and 100 μg/ml streptomycin, and 50 ng/ml m-CSF. After the cells were attached, the medium was replaced with m-CSF-depleted medium. The IncuCyte® Caspase-3/7 Green Apoptosis Assay Reagent coupling the activated caspase-3/7 recognition motif to NucView™488, was applicated according to the manufacturer’s instructions (Sartorius). Cells were imaged using a 20x objective with IncuCyte® live-cell analysis system every 3 h in total for 24 h. Cells seeded in replicate wells were incubated with 1 μM Vybrant™ DyeCycle™ Green Stain (Invitrogen) for 0.5 h and stained cell nuclei were imaged with 250 ms green channel acquisition time for counting cells. Images were analyzed using the IncuCyte™ Analysis Software. Release of the DNA dye and green fluorescent staining upon cleavage by activated Caspase-3/7 were monitored and averaged from four images taken per well. The data was normalized to the corresponding cell count. Green fluorescent area was normalized to the WT group at the latest timepoint (set as 100%) and shown as mean ± SEM.

### BMDM phagocytosis assay

For phagocytosis assays, 10,000–20,000 KI (*n* = 7) and WT (*n* = 6) BMDMs were plated on a 96 well plate 1 day prior to the assay. The assay was repeated in two independent experiments. Phagocytosis was initiated by adding 5 μg/well pHrodo-labeled bioparticles (pHrodo™ Red Zymosan BioParticles™ Conjugate for Phagocytosis, ThermoFisher Scientific, P35364) with or without 10 μM Cytochalasin D. Four fluorescent images per well with 300 ms red channel acquisition time were taken every 15 min in total for three-hours using a 20x objective with the IncuCyte® S3 Live-Cell Analysis System (Sartorius). At the end of the assay, cells were incubated with 1 μM Vybrant™ DyeCycle™ Green Stain (Invitrogen) for 0.5 h and stained cell nuclei were imaged with 250 ms green channel acquisition time for counting cells. Images were analyzed using the IncuCyte™ Analysis Software. pHrodo red fluorescence area averaged from four images taken per well were normalized to the corresponding cell count. Red fluorescent area was normalized to the WT group at the latest timepoint and shown as mean ± SEM.

### BV2 cell transfection and phagocytosis assay

pLenti-C-Myc-DDK (control) and human PLCγ2-myc-DDK (WT) in pLenti-C backbone vectors were obtained from OriGene (PS100064, RC200442L1). PLCγ2-myc-DDK was subjected to site-directed mutagenesis (QuikChange Lightning Multi Site-Directed Mutagenesis Kit, Agilent, 210,515) to create PLCγ2-P522R-myc-DDK (P522R) constructs. Briefly, the pLenti-PLCγ2-myc-DDK plasmid was amplified using a single mutagenic primer (5′-AGTGCCCCAGGATATACGCCCTACAGAACTAC-3′). Subsequently, the original plasmid was digested with DpnI restriction enzyme and the remaining mutagenized plasmid was transformed into XL10-Gold® ultracompetent cells (Agilent). Integrity of the insert sequence and introduction of the point mutation were confirmed by Sanger sequencing of the isolated plasmid DNA.

For phagocytosis assays, BV2 microglia cells were transfected using Viromer Yellow kit (Lipocalyx) according to the manufacturer’s instructions with small adjustments. In brief, 50,000 BV2 cells per well were seeded on a 12-well plate in RPMI 1640 (without phenol red) supplemented with 2 mM L-Glutamine, 1% (v/v), fetal calf serum (FCS), 100 U/ml penicillin, and 100 μg/ml streptomycin 1 day prior to the transfection. One μg of WT, P522R, or control (CTRL) plasmids together with 0,32 μl of Viromer Yellow in 100 μl of Viromer Yellow buffer per well were used for transfections. Transfection medium was replaced 4 h1 after transfections started. On the next day, cells were washed with PBS and incubated with pHrodo-labeled bioparticles (pHrodo™ Green *E. coli* BioParticles™ Conjugate for Phagocytosis, ThermoFisher Scientific) in OptiMem for 3 h at + 37 °C. Cells were then washed with PBS, gently scraped in flow cytometry buffer (1% FCS, 2 mM EDTA in PBS) and transferred to v-shaped 96-well plates. Non-viable cells were excluded by staining with 7-AAD Staining Solution (Abcam) for 5 min and pHrodo fluorescent signal was analyzed from 20,000 live cells by flow cytometry. Fluorescent signals were normalized to unstained (no pHrodo-labeled bioparticles) control within each group. Phagocytic activity is shown as geometric median of the fluorescence intensity (MFI) and as % of phagocytic cells within the analyzed cell population as mean ± SD. MFI was normalized to the WT group.

### TNFα, IL-6, and IL-1β ELISA and NO assay

To address acute inflammatory responses, one million KI (*n* = 4) and WT (*n* = 3) BMDMs were plated on six-well plates and allowed to attach. Cells were then treated with 1 μg/ml LPS and 20 ng/ml IFN-γ in PBS or with vehicle (PBS) for 3 h. Afterwards, media were collected and the levels of TNFα, IL-6, and IL-1β were determined using Mouse TNF alpha, IL-6, and IL-1 beta ELISA Ready-SET-Go!™ kits, respectively (Invitrogen™, eBioscience™). Cytokine levels were normalized to the total protein concentration in the lysate measured with BCA protein assay kit (Thermo Scientific). NO levels were analyzed using Griess Reagent Kit for Nitrite Determination (G-7921, Life Technologies) and normalized to the total protein concentration within the corresponding lysate. All kits were used as instructed by the manufacturers. Data are normalized to the WT BMDMs and presented as mean ± SD.

### NanoString gene expression analysis

Brains of the same six-month old KI and WT mice used for establishing the BMDM cultures were removed after transcardial perfusion and dissected into two hemispheres. Frozen right hemibrain was crunched in liquid nitrogen and 10-20 mg of pulverized mouse brain was used for RNA extraction with RNeasy Plus Mini Kit (Qiagen). RNA concentration and quality were determined using Agilent RNA 6000 Nano Kit according to the manufacturer’s recommendations. Gene expression data of the KI (*n* = 3) and WT (*n* = 3) mice were generated with the mouse neuropathology gene expression panel of the *NanoString* Technologies using the nCounter system. Gene expression data were analyzed using nSolver Advanced analysis software (*NanoString* Technologies) with build-in quality control, normalization, and statistical analyses. Expression data of significantly altered genes for individual animals are shown as Log2-transformed fold changes. Themes annotated with individual genes are derived from the *NanoString* Neuropathology panel. Pathway analysis was done using pathway annotations provided by the panel.

### RT-qPCR analysis

For RT-qPCR analysis, RNA was reverse-transcribed into cDNA using the SuperScript III First-Strand Synthesis System (Thermo Fisher Scientific) according to the manufacturer’s protocol. Target specific PCR primers for mouse *Cst7* (5′-GTGAAGCCAGGATTCCCCAA-3′ and 5′-GCCTTTCACCACCTGTACCA-3)*, Ccl3* (5′-CCAGCCAGGTGTCATTTTCC-3′ and 5′- AGTCCCTCGATGTGGCTACT-3′)*,* and *Ctsd* (5′-AATCCCTCTGCGCAAGTTCA-3′ and 5′- CGCCATAGTACTGGGCATCC-3′) were obtained from TAG Copenhagen. FastStart Universal SYBR Green Master (Roche) was used for qPCR. The comparative ΔΔCt method was used to calculate *Gapdh* (5′-CAGGAGAGTGTTTCCTCGTCC-3 and 5′-TTCCCATTCTCGGCCTTGAC-3′) -normalized expression levels of the target mRNAs. Expression of *Tyrobp* (Mm.PT.58.6069426, IDT), *Clec7a* (Mm.PT.58.42049707, IDT)*, and Plcγ2* (Mm01242530_m1, Thermo Fisher Scientific) were determined with mouse TaqMan assays and normalized to the expression of *Actb* (Mm.PT.39a.22214843.g, IDT) in the corresponding samples. Data are normalized to the expression levels in the WT group and shown as mean ± SD.

### Protein extraction and immunoblotting

Proteins were extracted from cultured cells or 20-40 mg of total mouse brain powder using RIPA buffer (150 mM NaCl, 10 mM Tris, pH 7.2, 0.1% SDS, 1% Triton X-100, 1% deoxycholate, 5 mM EDTA) supplemented with protease and phosphatase inhibitor cocktails (Thermo Fisher Scientific). After incubation for 30 min on ice, cell debris was removed by centrifuging for 10 min at 10000×g. Synaptosomes were extracted from 20 mg of total brain powder with Syn-PER reagent (Thermo Fisher) supplemented with protease and phosphatase inhibitor cocktails (Thermo Fisher Scientific) according to the manufacturer’s instructions. Protein concentration was determined using the Pierce BCA Protein Assay Kit (Thermo Fisher Scientific). Proteins in NuPAGE LDS Sample Buffer were separated on NuPAGE 4–12% BisTris Mini or Midi Protein Gels (Invitrogen). SeeBlue Plus2 Pre-stained Protein Standard (Invitrogen) was used for sizing of proteins. Proteins were blotted onto a PVDF membrane using an iBlot 2 Gel Transfer Device (Thermo Fisher Scientific). The following antibodies were used to probe the blots at 4 °C overnight: anti-rabbit phospho-Akt (pan, Ser473/474, 1:1000, Cell Signaling Technologies), anti-rabbit Akt (pan, 1:1000, Cell Signaling Technologies), anti-mouse phospho-Erk1/2 (Thr202/Tyr204, 1:500, Santa Cruz Biotechnology), anti-mouse Erk2 (1:500, Santa Cruz Biotechnology), anti-rabbit phospho-Gsk3β (Ser9, 1:1000, Cell Signaling Technologies), anti-rabbit Gsk3β (1:1000, Cell Signaling Technologies), anti-rabbit LC3B (1:2000, Abcam), anti-mouse Myc (1:1000, Millipore), anti-rabbit Plcγ2 (1:1000, Cell Signaling Technologies), anti-mouse Plcγ2 (B-10, 1:500, Santa Cruz Biotechnology), anti-mouse Psd-95 (1:2000, ThermoFisher Scientific), anti-mouse Synaptophysin 1 (1:5000, SYSY), mouse anti-Gapdh (1:10000. Abcam), anti-mouse β-actin (1:1000, Abcam), or anti-mouse β-tubulin (1:2000, Millipore). The blots were incubated with respective HRP-conjugated anti-mouse or anti-rabbit secondary antibodies (1:5000, GE Healthcare) for 1 h at room temperature and detected using ECL Select Western Blotting Detection Reagent (GE Healthcare) and a ChemiDoc Imaging System (Bio-Rad). Protein levels were quantified with Image Lab Software 6.0.1 (Bio-Rad). Levels of the phosphoproteins were normalized to the corresponding total proteins and total proteins to the levels of Gapdh, β-actin or β-tubulin within the same sample. Data are shown percent of the WT group (set to 100%) and shown as mean ± SD.

### Immunofluorescence analyses and confocal imaging

Following cardiac perfusion, left-brain hemispheres were immerse-fixed in 4% paraformaldehyde for 24 h, and in 30% sucrose for 24 h subsequently. After freezing, 50 μm microtome cut free floating coronal sections were washed briefly and then blocked using 5% donkey serum for 1 h at room temperature. Following this, sections were incubated with primary antibodies (IBA1 1:300, In vitrogen; GFAP 1:500, Thermo Fisher Scientific) at 4 °C and (P2RY12 1:100, Biolegend) at room temperature overnight. Sections were washed and incubated in secondary antibodies (donkey anti-rabbit 1:500, donkey anti-rat 1:500, donkey anti-goat 1:1000) or 2 h at room temperature. Lastly, slides were washed and stained with 4′,6-Diamidin-2-phenylindol (DAPI, 5 μg/mL) before mounting coverslips with ProlongTM Gold Antifade reagent (Thermo Fisher Scientific). Images were acquired using a LSM 710 confocal microscope (Zeiss) and the ZEN 2011 software package (black edition, Zeiss). Laser and detector settings were maintained constant for the acquisition of each immunostaining. For analyses, an average of three sections per animal were used. At least three images were taken per slide to cover most of the cortex. Images were taken with10x (Plan-Apochromat 10x/0.45 M27), 20x (Plan-Apochromat 20x/0.8 M27), and 40x (Plan-apochromat 40x/1.4 Oil DIC M27) objectives Microglia cell numbers were counted on FIJI software (ImageJ) using the “Cell counter” plugin. For GFAP coverage analysis, confocal acquired images for cortex or hippocampus were imported to FIJI, and channels were separated by “Image/Color/Split Channels”. Following this, background noise was removed using Gaussian filtering and intensity distribution for each image was equalized using rolling ball algorithm. All layers from a single image stack were projected on a single slice by “Stack/Z projection”. Lastly, GFAP-positive staining was segmented using automatic thresholding method “Moments” in FIJI. Microglia morphology analysis was performed using FIJI software (ImageJ) and the AnalyzeSkeleton (2D/3D) plugin as previously described (Young & Morrison, 2018 J. Vis. Exp.). Z-stacks were imported into the software and converted to 8-bit grayscale max projections. After running the Unsharp Mask filter, a Despeckle step was performed to remove noise. The image was converted to binary by adjusting threshold and Despeckle, Close as well as Remove Outlier functions were performed, respectively. Lastly, the image was skeletonized to subsequently run the AnalyzeSkeleton (2D/3D) plugin. Four animals per genotype were used in the analysis. For each animal, an average of three slides were used for analyzing the cortex or hippocampus.

### PET-imaging

For PET imaging, one-year-old KI (*n* = 3) and WT (*n* = 4) female mice were anesthetized with isoflurane (1.5% with N2/O2 70%/30% through nose cone). The mice were placed on a heated animal holder on the scanner bed in a prone position and secured with tape to prevent movement during scanning. The mice were imaged using a dedicated PET scanner (Inveon DPET, Siemens Healthcare) and immediately afterwards with CT (Flex SPECT/CT, Gamma Medica, Inc.) for anatomical reference images using the same animal holder. Dynamic imaging of 70 min was started at the time of the administration of the activity (18F-FEPPA: 15.8 ± 1.4 MBq and 18F-FDG: 13.9 ± 1.2 MBq) through the tail vein. Data were gathered in list-mode form, and corrected for dead-time, randoms, scatter and attenuation. Regions of interest (ROIs) were drawn for whole brain (excluding cerebellum and olfactory bulb), cerebellum, pons, hippocampus and frontal cortex using Carimas 2.10 software (Turku PET Centre, Finland). Also, for image derived input function (IDIF) a ROI was drawn for the *veca cava*. Logan analysis for 18F-FEPPA-data and Patlak analysis for 18F-FDG data was done using the IDIF to estimate the inflammation and cerebral glucose metabolism respectively for the brain ROIs.

### Statistical analyses

Statistical significance between groups was tested using independent samples *t-*test or Mann-Whitney U test depending whether the data fulfilled the assumptions for parametric tests or with two-way ANOVA (more than two groups) followed by LSD post-hoc test. Gene expression data were analyzed using nSolver Advanced analysis software (NanoString Technologies) with build-in quality control, normalization, and statistical analyses. Immunohistochemical data were checked for normality using the Shapiro-Wilk method, the D’Agostino and Pearson, as well as the Kolmogrov-Smirnov normality tests. Statistical significance was calculated using two-tailed unpaired *t*-test. All statistical analyses were performed using IBM SPSS Statistics 25 or GraphPad Prism software. A threshold for statistical significance was set at *p* < 0.05.

## Supplementary information

**Additional file 1: Supplementary Figure 1.** Representative Sanger-sequencing chromatograms of the Plcγ2 on-target site and the seven putative off target sites of a heterozygous F1 animal. Mixed peaks in the Plcγ2 locus show the correct P522R substitution (CCC > AGG, on complementary strand) and a silent mutation for genotyping purposes. Underlined: Protospacer; arrowhead: putative cut site; green letters: PAM site on shown strand; red letters: PAM site on complementary strand.

**Additional file 2: Supplementary Figure 2.** A) Immunoblot showing protein levels of Plcγ2 in Plcγ2-P522R knock-in (KI) and wild type (WT) bone marrow-derived macrophages (BMDMs. Quantitation showing Gapdh-normalized Plcγ2 protein levels in WT and KI BMDMs. Mean ± SD, % of WT, *n* = 3 per genotype. Independent samples t-test. B) Caspase-3/7 activation in WT and KI BMDMs after macrophage colony stimulation factor 1 (mCSF) withdrawal during 24 h. Four images were taken per well with a 20x objective every 4 h. Signals were normalized to the number of nuclei in the corresponding samples. Mean ± SEM, % of WT (24 h), *n* = 3 per genotype, 4 technical replicates. Independent samples t-test. C) Area under the curve (AUC) analysis indicating overall difference in phagocytic activity between WT and KI BMDMs. Mean ± SEM, % of WT, *n* = 6–7 per genotype, 3 technical replicates. Independent samples t-test. D) Immunoblot showing overexpression of Myc-tagged human PLCγ2-WT and PLCγ2-P522R constructs in BV2-cells using anti-Plcγ2 and anti-Myc antibodies. Quantitation showing β-actin-normalized PLCγ2 levels. Mean ± SD, % of CTRL, *n* = 4. Independent samples T-test, *p < 0.05, ***p < 0.001.

**Additional file 3: Supplementary Figure 3.** A) Heat map showing all significantly (*p* < 0.05) changed genes and associated themes obtained from the neuropathology panel. Targets are arranged according to log2-transformed fold-changes in Plcγ2-P522R homozygote knock-in (KI) mice as compared to the wild type (WT) littermates, *n* = 3 per genotype. B) String-network graphic and kmeans clustering of significantly affected targets associated with *Plcγ2* (https://string-db.org/cgi/network.pl?taskId=AF3kTOpi6ovG). C) RNA expression levels of *Plcγ2* and microglia specific disease-associated microglia signature genes, *Cst7*, *Tyropb, Clec7a, Ccl3*, and *Ctsd* in the brain of six-month-old Plcγ2-P522R homozygous knock-in (KI) and wild type (WT) mice. Normalized to the WT group, mean ± SD, *n* = 3–4 per genotype.

**Additional file 4: Supplementary Figure 4.** A) Immunoblot showing the levels of phosphorylated Gsk3β at S9, total Gsk3β, and LC3BI, and LC3BII in total brain lysate of Plcγ2-P522R knock-in (KI) and wild type (WT) mice at 6-month of age. Respective quantitation showing total protein normalized phospho-protein levels, Gapdh-normalized total protein levels and the ratio of LC3BII/I. Mean ± SD, % of WT, *n* = 3–4 per genotype. Independent samples T-test, *p < 0.05. B) Immunoblot showing cytosolic and synaptosomal extracts obtained from total brain lysates of 6-month old WT and KI mice. Quantitation showing β-Tubulin normalized levels of pre- and post-synaptic markers Synaptophysin and Psd-95 in the synaptosomal fraction. Mean ± SD, % of WT, *n* = 4 per genotype. Independent samples T-test.

## Data Availability

All data generated or analyzed during this study are included in this published article and its supplementary information files.

## Abbreviations

Plcγ2: Phospholipase C gamma 2
Pip2: Phosphatidylinositol 4,5-bisphosphate
Ip3: 1,4,5-trisphosphate
Dag: Diacylglycerol
Trem2: Triggering receptor expressed on myeloid cells 2
Nfκb: Nuclear factor kappa-light-chain-enhancer of activated B cells
Mapk/Erk: Mitogen-activated protein kinase
Akt: Protein kinase B
KI: Knock-in
WT: Wild type
BMDM: Bone marrow-derived macrophage
iMG: Induced microglia-like cells
Ip1: Inositol monophosphate
LPS: Lipopolysaccharide
IFNγ: Interferon gamma
TNFα: Tumor necrosis factor alpha
IL-6: Interleukin-6
IL-1β: Interleukin 1 beta
NO: Nitric oxide
DAM: Disease associated microglia
