# Supplementary figures and images for: The Alzheimer’s disease-associated protective Plcγ2-P522R variant promotes immune functions
Source: Mol Neurodegener. 2020 Sep 11;15:52. doi: 10.1186/s13024-020-00402-7 (PMC7488484; doi:10.1186/s13024-020-00402-7)

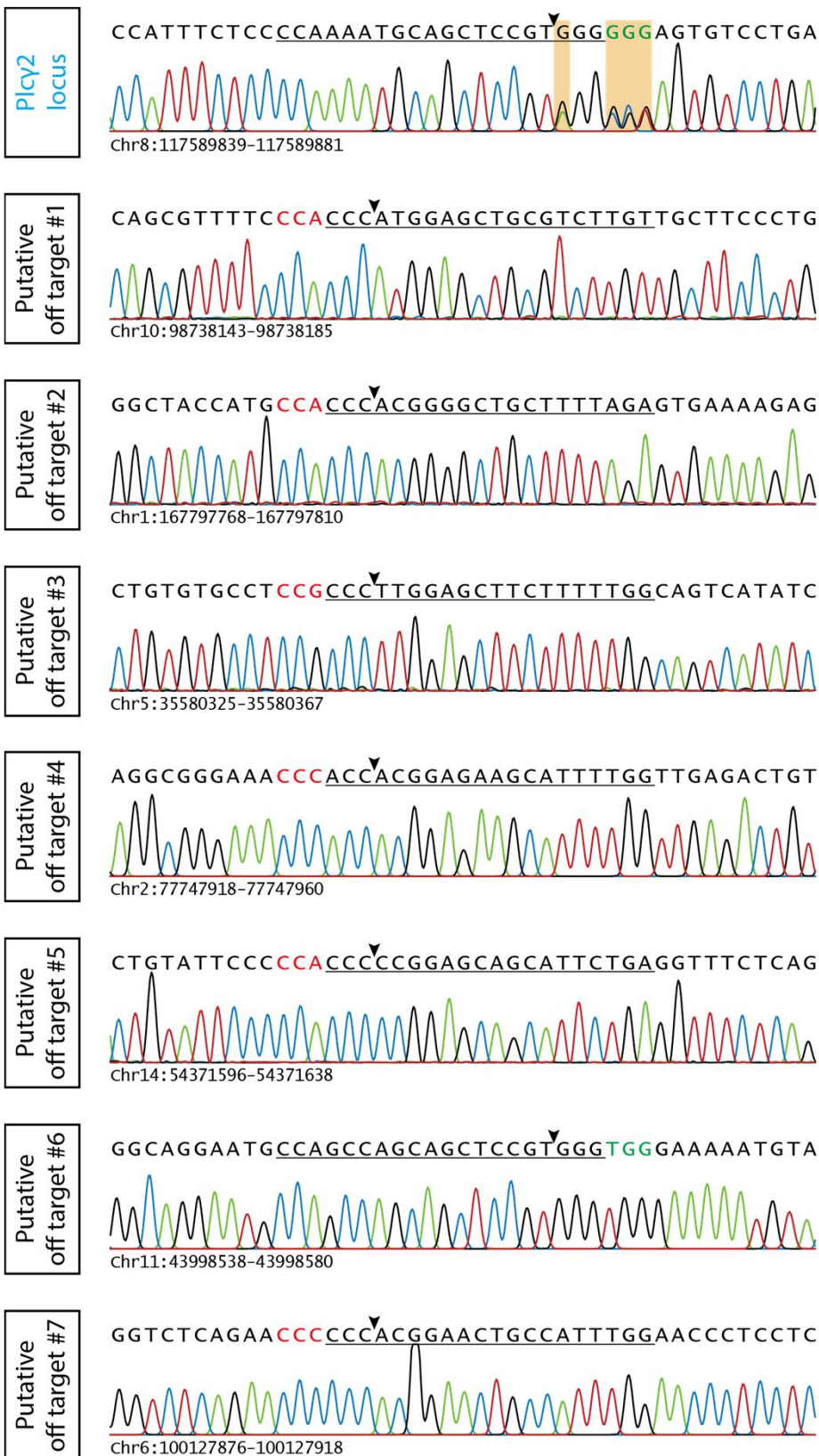

Supplement: Supplementary file 1 — Additional file 1: Supplementary Figure 1. Representative Sanger-sequencing chromatograms of the Plcγ2 on-target site and the seven putative off target sites of a heterozygous F1 animal. Mixed peaks in the Plcγ2 locus show the correct P522R substitution (CCC > AGG, on complementary strand) and a silent mutation for genotyping purposes. Underlined: Protospacer; arrowhead: putative cut site; green letters: PAM site on shown strand; red letters: PAM site on complementary strand. [file 13024_2020_402_MOESM1_ESM.pdf]

**A.**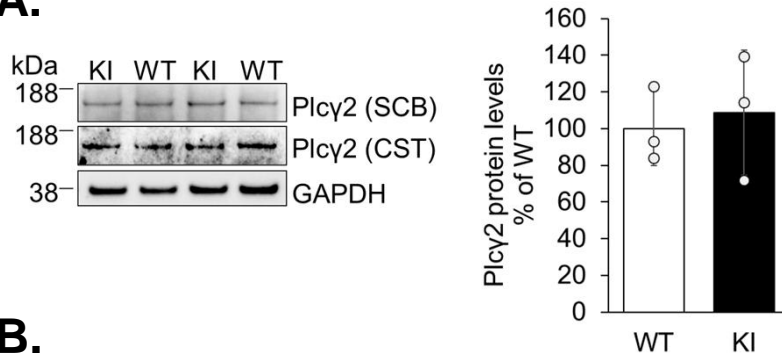**B.**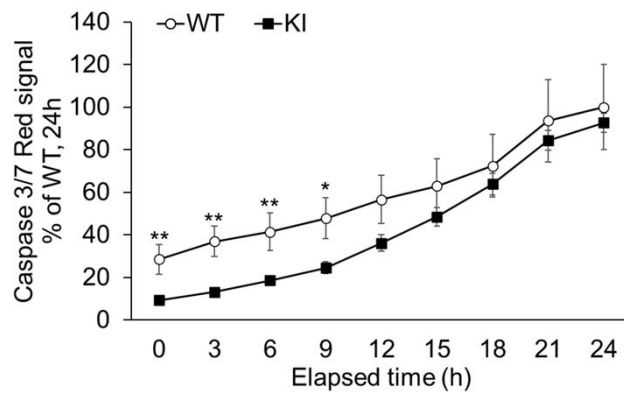**C.**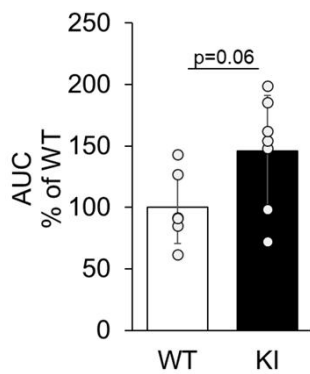**D.**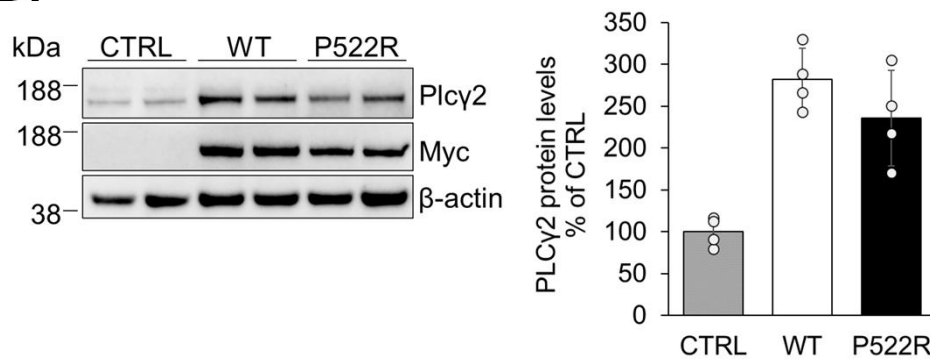

Supplement: Supplementary file 2 — Additional file 2: Supplementary Figure 2. A) Immunoblot showing protein levels of Plcγ2 in Plcγ2-P522R knock-in (KI) and wild type (WT) bone marrow-derived macrophages (BMDMs. Quantitation showing Gapdh-normalized Plcγ2 protein levels in WT and KI BMDMs. Mean ± SD, % of WT, n = 3 per genotype. Independent samples t-test. B) Caspase-3/7 activation in WT and KI BMDMs after macrophage colony stimulation factor 1 (mCSF) withdrawal during 24 h. Four images were taken per well with a 20x objective every 4 h. Signals were normalized to the number of nuclei in the corresponding samples. Mean ± SEM, % of WT (24 h), n = 3 per genotype, 4 technical replicates. Independent samples t-test. C) Area under the curve (AUC) analysis indicating overall difference in phagocytic activity between WT and KI BMDMs. Mean ± SEM, % of WT, n = 6–7 per genotype, 3 technical replicates. Independent samples t-test. D) Immunoblot showing overexpression of Myc-tagged human PLCγ2-WT and PLCγ2-P522R constructs in BV2-cells using anti-Plcγ2 and anti-Myc antibodies. Quantitation showing β-actin-normalized PLCγ2 levels. Mean ± SD, % of CTRL, n = 4. Independent samples T-test, *p < 0.05, ***p < 0.001. [file 13024_2020_402_MOESM2_ESM.pdf]

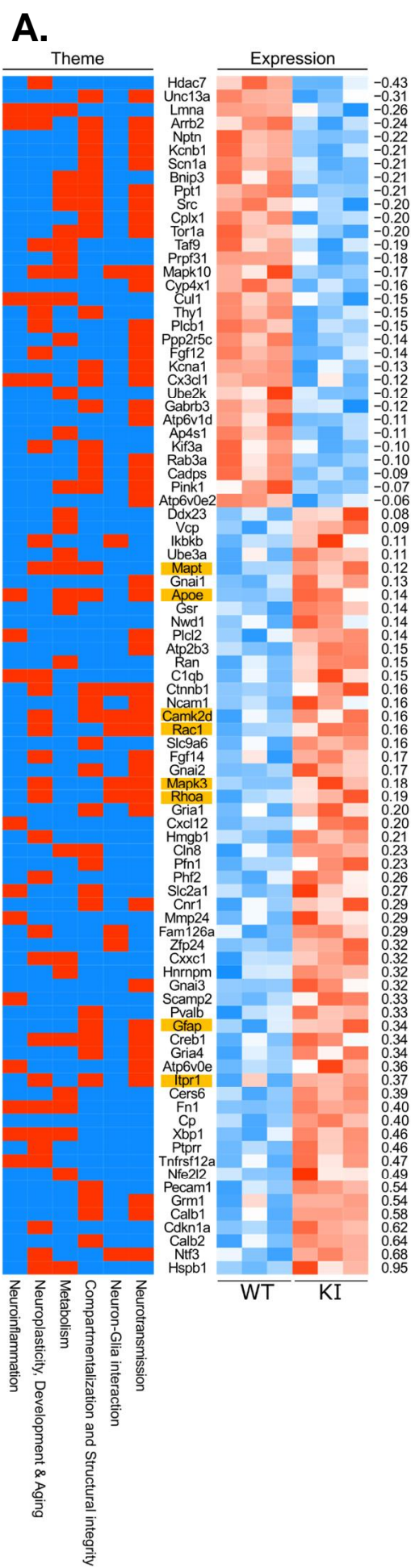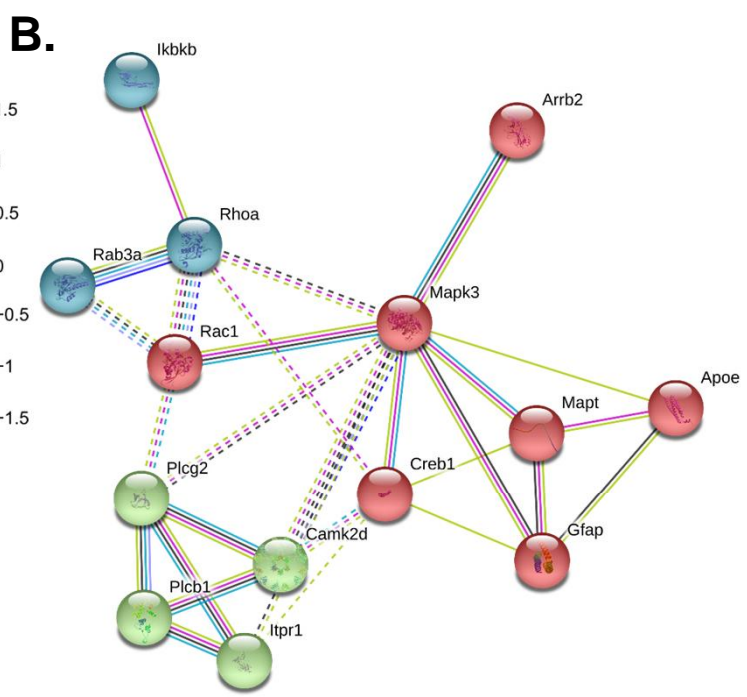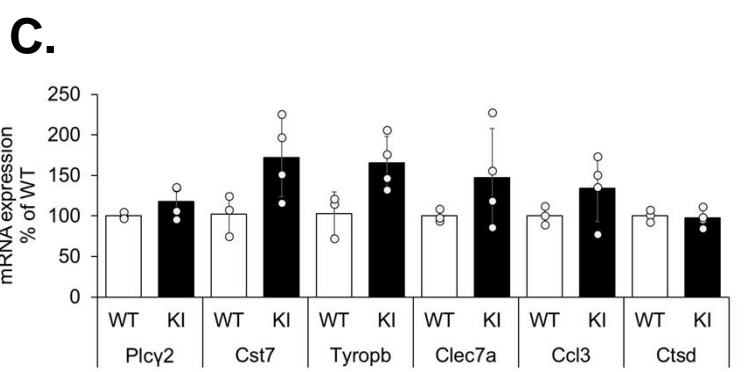

Supplement: Supplementary file 3 — Additional file 3: Supplementary Figure 3. A) Heat map showing all significantly (p < 0.05) changed genes and associated themes obtained from the neuropathology panel. Targets are arranged according to log2-transformed fold-changes in Plcγ2-P522R homozygote knock-in (KI) mice as compared to the wild type (WT) littermates, n = 3 per genotype. B) String-network graphic and kmeans clustering of significantly affected targets associated with Plcγ2 (https://string-db.org/cgi/network.pl?taskId=AF3kTOpi6ovG). C) RNA expression levels of Plcγ2 and microglia specific disease-associated microglia signature genes, Cst7, Tyropb, Clec7a, Ccl3, and Ctsd in the brain of six-month-old Plcγ2-P522R homozygous knock-in (KI) and wild type (WT) mice. Normalized to the WT group, mean ± SD, n = 3–4 per genotype. [file 13024_2020_402_MOESM3_ESM.pdf]

**A.**

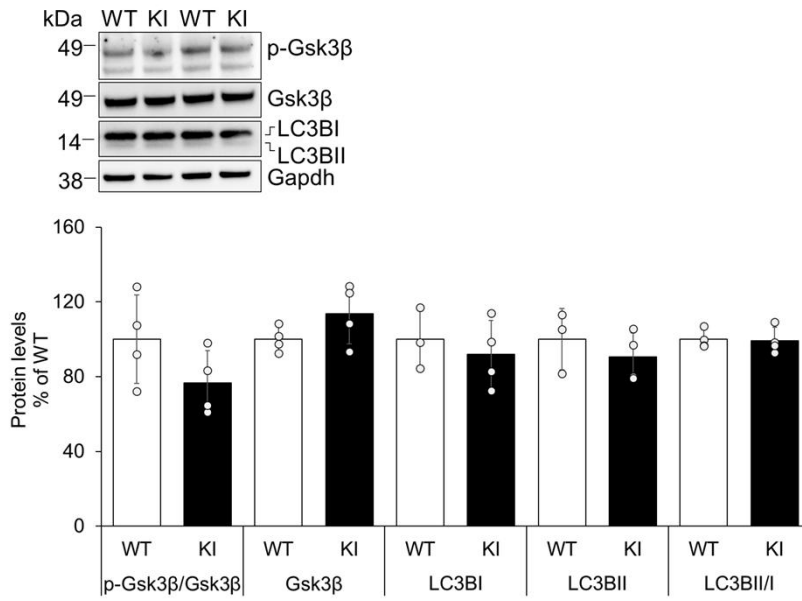

**B.**

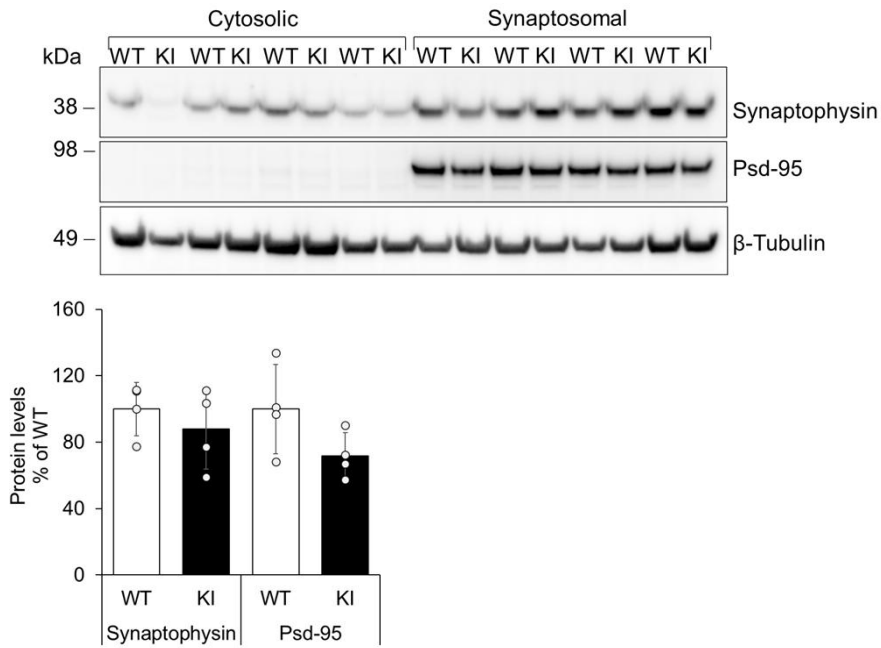

Supplement: Supplementary file 4 — Additional file 4: Supplementary Figure 4. A) Immunoblot showing the levels of phosphorylated Gsk3β at S9, total Gsk3β, and LC3BI, and LC3BII in total brain lysate of Plcγ2-P522R knock-in (KI) and wild type (WT) mice at 6-month of age. Respective quantitation showing total protein normalized phospho-protein levels, Gapdh-normalized total protein levels and the ratio of LC3BII/I. Mean ± SD, % of WT, n = 3–4 per genotype. Independent samples T-test, *p < 0.05. B) Immunoblot showing cytosolic and synaptosomal extracts obtained from total brain lysates of 6-month old WT and KI mice. Quantitation showing β-Tubulin normalized levels of pre- and post-synaptic markers Synaptophysin and Psd-95 in the synaptosomal fraction. Mean ± SD, % of WT, n = 4 per genotype. Independent samples T-test. [file 13024_2020_402_MOESM4_ESM.pdf]
